# Neutrophil Extracellular Traps Induce the Epithelial-Mesenchymal Transition: Implications in Post-COVID-19 Fibrosis

**DOI:** 10.3389/fimmu.2021.663303

**Published:** 2021-06-14

**Authors:** Laura Pandolfi, Sara Bozzini, Vanessa Frangipane, Elena Percivalle, Ada De Luigi, Martina Bruna Violatto, Gianluca Lopez, Elisa Gabanti, Luca Carsana, Maura D’Amato, Monica Morosini, Mara De Amici, Manuela Nebuloni, Tommaso Fossali, Riccardo Colombo, Laura Saracino, Veronica Codullo, Massimiliano Gnecchi, Paolo Bigini, Fausto Baldanti, Daniele Lilleri, Federica Meloni

**Affiliations:** ^1^ Research Laboratory of Lung Diseases, Section of Cell Biology, IRCCS Policlinico San Matteo Foundation, Pavia, Italy; ^2^ Molecular Virology Unit, Microbiology and Virology Department, IRCCS Policlinico S. Matteo Foundation, Pavia, Italy; ^3^ Laboratory of Biochemistry and Protein Chemistry, Department of Biochemistry and Molecular Pharmacology, Istituto di Ricerche Farmacologiche “Mario Negri” IRCCS, Milano, Italy; ^4^ Pathology Unit, ASST Fatebenefratelli Sacco, Luigi Sacco Hospital, University of Milano, Milano, Italy; ^5^ Biochemistry Unit, Department of Molecular Medicine, University of Pavia, Pavia, Italy; ^6^ Laboratory of Immuno Allergology Clinical Chemistry and Pediatrics Clinic, Foundation IRCCS Policlinico San Matteo, University of Pavia, Pavia, Italy; ^7^ Division of Anaesthesiology and Intensive Care, ASST Fatebenefratelli Sacco, Luigi Sacco Hospital, University of Milan, Milan, Italy; ^8^ Unit of Pneumology, IRCCS Policlinico San Matteo Foundation, Pavia, Italy; ^9^ Unit of Rheumatology, IRCCS Policlinico San Matteo Foundation, Pavia, Italy; ^10^ Coronary Care Unit and Laboratory of Clinical and Experimental Cardiology, Fondazione IRCCS Policlinico San Matteo, Pavia, Italy; ^11^ Department of Molecular Medicine, Cardiology Unit, University of Pavia, Pavia, Italy; ^12^ Department of Internal Medicine, University of Pavia, Pavia, Italy; ^13^ Department of Internal Medicine, Policlinico San Matteo Foundation, Pavia, Italy

**Keywords:** NETosis, SARS-CoV2, COVID-19, epithelial-mesenchymal transition, lung fibrosis

## Abstract

The release of neutrophil extracellular traps (NETs), a process termed NETosis, avoids pathogen spread but may cause tissue injury. NETs have been found in severe COVID-19 patients, but their role in disease development is still unknown. The aim of this study is to assess the capacity of NETs to drive epithelial-mesenchymal transition (EMT) of lung epithelial cells and to analyze the involvement of NETs in COVID-19. Bronchoalveolar lavage fluid of severe COVID-19 patients showed high concentration of NETs that correlates with neutrophils count; moreover, the analysis of lung tissues of COVID-19 deceased patients showed a subset of alveolar reactive pneumocytes with a co-expression of epithelial marker and a mesenchymal marker, confirming the induction of EMT mechanism after severe SARS-CoV2 infection. By airway *in vitro* models, cultivating A549 or 16HBE at air-liquid interface, adding alveolar macrophages (AM), neutrophils and SARS-CoV2, we demonstrated that to trigger a complete EMT expression pattern are necessary the induction of NETosis by SARS-CoV2 and the secretion of AM factors (TGF-β, IL8 and IL1β). All our results highlight the possible mechanism that can induce lung fibrosis after SARS-CoV2 infection.

## Introduction

Neutrophils represent the most abundant type of white blood cells. Neutrophils protect against foreign pathogens and are considered an essential component of the innate immune system. After activation, neutrophils can react against pathogens with three major different mechanisms: i) phagocytosis; ii) release of granules (that contain proteases and ROS); iii) NETosis. This last mechanism is considered a type of programmed neutrophil cell death, which lead to the release of neutrophil extracellular traps (NETs) (1, 2). NETs are web-like structures composed of chromatin decorated with proteases, such as human neutrophils elastase (HNE) and myeloperoxidase (MPO), whose primary role is limiting the spread of pathogens in tissues (3). Several signals can stimulate NETosis: microorganisms; pro-inflammatory cytokines (IL8 and IL1β), and chemicals (PMA) (4).

In spite of their protective role in reducing pathogens diffusion in the parenchyma, several studies revealed that persistence of NETs can amplify the primary injury inducing further clinical complications (2, 5, 6). In the lung, the persistence of NETs has been linked to several diseases, such as cystic fibrosis and acute respiratory distress syndrome (ARDS). Notably, COVID-19 patients who developed ARDS show increased NETs in the serum and, most importantly, NETs release significantly correlates with the severity of the lung pathology (7). Moreover, a recent paper demonstrated that NETs are detectable in tracheal aspirate of patients with COVID-19 and that they are involved in the prothrombotic clinical manifestations of COVID-19 (8). The direct consequence of NETs persistence in the lung is the damage of epithelial and endothelial cells, driven predominantly by histones (1). Additionally, NETs have been found to drive the EMT in the context of the breast cancer due to their capacity to upregulate transcription factors involved in the EMT (ZEB1 and SNAI1) (9).

Given these premises, the aim of this study is to assess whether NETosis may drive the EMT also in lung epithelial cells. This would represent a new crucial molecular mechanism underlying the development of inflammatory-induced lung fibrosis, thus it would also be of great importance for several pulmonary diseases, such as autoimmune microbial insults and transplant rejection. Moreover, this study is aimed at understanding if NETs could be implicated in the induction of EMT in SARS-CoV2 pneumonia promoting fibrosis.

## Material and Methods

### COVID-19 Patients

Bronchoalveolar lavage (BAL) of 33 adults positive for SARS-CoV2 infection, diagnosed by real-time PCR on nasopharyngeal swab, were collected. N = 28 were admitted to the Intensive Care Unit (ICU) at the Luigi Sacco Hospital (Milan, Italy); N = 5 were admitted to the Intermediate Medicine ward (IMW) of the IRCCS Policlinico San Matteo Foundation. Failure of a trial with continuous positive airway pressure *via* a helmet was the indication for admission in ICU. Failure was defined as respiratory rate >30 breaths per minute and PaO_2_ to FiO_2_ ratio <150, or respiratory acidosis with pH<7.36 and PaCO_2_ >50 mmHg, or agitation, or confusion. BALs method collection and demographic characteristics of the studied population can be found in the already published paper (10).

Two of collected BAL was cytospinned to produce spots that were stained using Papanicolaou, May-Grunwald-Giemsa and Ziehl-Neelsen methods. The remaining material was fixed in 10% buffered formalin and routinely processed to form cell-blocks. From the cell-blocks, 3-μm paraffin sections were stained by H&E for cytological examination.

Lung autopsy tissues from two patients died of SARS-CoV2 pneumonia were fixed in 10% buffered formalin for 48 h. Three-μm paraffin sections were stained by H&E. Immunohistochemistry reactions were performed on the most representative area by using anti-Cytokeratin 7 (CK7) (clone SP52, Ventana) and anti-α-SMA (clone 1A4, Ventana).

### Cell Culture

A549 and 16HBE cell line (purchased from ATCC^®^) was cultivated in DMEM high glucose supplemented with 10% of FBS, 1% of penicillin-streptomycin solution and 1% of L-glutamine (all purchased by EuroClone). Cell were cultivated at 37°C with 5% of CO_2_ and harvested when they reached the 80% of confluence.

### Immune Cells Isolation for *In Vitro* Airway Model

Neutrophils were isolated from peripheral blood of healthy donors. Blood was stratified by Lympholyte^®^ Cell Separation Media (EuroClone, Milan, Italy) and after centrifugation at 450 g for 30 min mononuclear cell phase was eliminated to allow neutrophils isolation. A solution of 3% Dextran (200 kDa) in 0.85% NaCl was added to the remaining phases of neutrophils and erythrocytes followed by a 30 min incubation at room temperature, allowing the precipitation of erythrocytes. Supernatant was collected and washed with PBS (EuroClone). After centrifugation at 450 g for 10 min, pellet was resuspended with VersaLyse Lysing Solution (Beckman Coulter S.r.l., Milano) for 20 min at room temperature in the dark. After the addition of PBS, sample was centrifuged again at 450 g for 10 min. This passage was repeated until the pellet was found to be free of erythrocytes.

Regarding macrophages, we decided to use alveolar macrophages (AM) isolated from BAL of patients subjected to bronchoscopy for diagnosis purpose, that did not show any sign of lung infection and with a cytologic count of AM > 76% and lymphocytes < 14%. BAL were filtered with a sterile gauze, centrifuged at 450 g for 10 min and then cell pellet were cultured in suspension for 24 h in DMEM containing 10% FCS, P/S and L-glutamine.

### NETosis, NETs Isolation and Quantification

To induce the release of NETs and to isolate them we followed a published protocol with some modifications (11). 2.5 x 10^6^ neutrophils were cultivated in 60 cm^2^ plate with the addition of 100 nM PMA (Sigma-Aldrich S.r.l., Milan, Italy) for 4 h at 37°C (PMA-Neu). After that, supernatant was discarded, and the layer of NETs present onto the bottom of the plate was harvested using PBS and collected into falcon. After centrifugation at 450 g for 10 min, cell-free NETs-rich supernatant was divided in 1.5 mL Eppendorf and centrifuged at 18,000 g for 10 min at 4°C.

For confocal microscopy, 2 x 10^6^ PMA-Neu or non-activated neutrophils were washed with PBS and fixed with 4% of paraformaldehyde for 10 min. After three washes with PBS, cells were treated with blocking solution (1% BSA in PBS) for 1 h at room temperature. Cell were than stained with anti-H3 polyAb (dilution 1:100 – PA5-31954 - Invitrogen - Life Technologies) or anti-MPO polyAb (dilution 1:50 – DOM0001G - Invitrogen) or anti-HNE mAb (dilution 1:50 – MA1-40220 - Invitrogen) in 0.5% BSA in PBS for 1 h at room temperature. After three washes in PBS, secondary mAbs (anti-mouse and anti-rabbit IgG H&L, Alexa Fluor^®^ 488 - Abcam; anti-rabbit IgG H&L, Alexa Fluor^®^ 647 - Invitrogen) was added in 0.5% BSA in PBS for 1 h at room temperature. Neutrophils without treatment with PMA were used as control. Coverslips were than mounted using ProLong™ Gold Antifade Mountant with DAPI (Invitrogen) and analyzed with confocal microscopy (Fluoview FV10i, Olympus).

To quantify NETs, we measured free DNA using Cell Death Detection ELISAPLUS kit (Roche) and Citrullinated Histone H3 (CitH3) (Clone 11D3) ELISA Kit (Cayman).

### EMT Induction in Monoculture

To study the induction of EMT on A549 by PMA-Neu or pure NETs, 0.2 x 10^6^ A549 were cultured on 12-well plate for 24 h. Then, 2.5 x 10^6^ PMA-Neu or pure NETs derived from the same number of cells were added in each well and after 24 h supernatants were discarded and A549 were lysed to extract all proteins or total RNA to perform western blot and real-time PCR (RT-PCR) analysis. As positive control we treated A549 with 10 µg mL^-1^ of TGF-β.

Cell death was evaluated labeling A549 with propidium iodide (PI) after 24 h of incubation with 100 nM PMA, 2.5 x 10^6^ PMA-Neu or NETs derived from the same number of cells and analyzed with flow cytometry.

### SARS-CoV2

A wild strain of SARS-CoV2 was isolated in VERO E6 cell line from a nasal swab of a COVID-19 infected patient. Virus was propagated and titrated to prepare a stock to be stored at –80°C to be used for all the experiments at a concentration of 100 TCID50. The amount of 100TCID50 was chosen on the basis of virus titration previously performed according to the procedure reported (12).

### Airway *In Vitro* Model

To mimic the airway microenvironment *in vitro*, 0.5 x 10^6^ A549 cells were cultured onto transwell inserts (0.4 µm-pore size - Corning Costar) in air liquid interface (ALI) to allow a good polarization of cells. After 14 days, different conditions were prepared:

Only A549 as control

A549 + SARS-CoV2 (added onto A549 in 20 µL to not modify the polarization of cells)

A549 + 2.5 x 10^6^ neutrophils + SARS-CoV2

A549 + 0.5 x 10^6^ AM + SARS-CoV2

A549 + 0.5 x 10^6^ AM + 2.5 x 10^6^ neutrophils + SARS-CoV2

After 48 h, medium in the lower compartment was collected to perform cytokines/NETs quantifications, transwell inserts were cut around the membrane edges to lyse cells to extract all proteins or total RNA.

### Western Blot

Cells were lysed with lysis buffer (50 mM Tris-HCl [pH 7.4], 150 mM NaCl, 10% glycerol, 1% NP-40, protease inhibitor cocktail (Sigma Aldrich) and phosphatase inhibitor (Roche)), gently vortexed for 20 min at 4°C and centrifuged for 15 min at 13,200 rpm at 4°C. Supernatants were quantified by Pierce™ BCA Protein Assay Kit (Thermo Fisher Scientific).

Twenty micrograms of proteins from A549 cell extracts were loaded and separated in 8% SDS-PAGE. After electrophoresis, the gels were transferred to polyvinylidene difluoride membranes (Millipore), therefore blocked (5% no fat milk in 0.1% Tween 20 TBS) and incubated with the primary Ab (1:1000 in TBST + 2% BSA; overnight at 4°C or 2 h at room temperature): anti-E-Cadherin [M168] (ab76055, Abcam), anti-α-SMA [E184] (ab32575, Abcam), and anti-β-Actin (MAB1501R, Chemicon). After wash, the membranes were incubated with the appropriate HRP-conjugated secondary Ab (1:5000 in TBST + 2% BSA; 2 h at room temperature; anti-mouse A4416 and anti-rabbit A0545, Sigma). The immunoreactivity was detected by ECL reagents (Amersham), acquired with the ChemiDoc imaging system (Image Lab, Bio-Rad).

### RT-PCR

Total RNA was isolated from cells using miRNeasy Mini Kit (Qiagen). RNA concentration and purity were evaluated using a spectrophotometer (Nanodrop 2000, Thermo Scientific, Madison, WI, USA). cDNA was retrotranscribed from 1 µg of total RNA using LunaScript RT SuperMix Kit (NEB), according to the manufacturer’s instructions. Relative levels of Alpha Smooth Muscle Actin (ACTA2) and E-Cadherin (CDH1) mRNA were assessed using SYBR^®^ Green Luna^®^ Universal qPCR Master Mix (NEB) and normalized to the levels of glyceraldehyde-3- phosphate dehydrogenase (GAPDH) mRNA. All reactions were performed on an LC480 Real-Time PCR system (Roche Diagnostics, Vienna, Austria) according to the manufacturer’s recommendations. Each experiment was performed in triplicate. The threshold cycle (Ct) was defined as the fraction cycle number at which fluorescence exceeded the given threshold. Relative gene expression level quantification was compared with internal standards and analyzed using the 2^−∆∆Ct^ method.

### Cytokines Quantification

To quantify cytokines released in the alveolar *in vitro* model, ELISA assays were performed. We quantified IL8 with SimpleStep ELISA^®^ Kit (Abcam), TGF-β (Abcam) and IL1β with human IL-1β/IL-1F2 Immunoassay (R&D Systems) was titered using a commercial enzyme-linked immunosorbent assay kit (Human IL-1β/IL-1F2 Immunoassay, R&D Systems) following the manufacturer’s instructions and the results were expressed as pg ml^-1^. All determinations were measure in same session. For IL8 quantification in BAL of COVID-19 patients we referred to already published quantifications (10).

### Statistical Analysis

*In vitro* results presented in this study were analyzed by one-way ANOVA followed by Dunnet test, or by unpaired t-test. Results were obtained from three/four independent experimental replicates represented as mean ± SD. BAL analyses were done by Mann-Whitney test, while correlation analyses were done calculating Spearman coefficient. Data are represented as median (interquartile range – IQR). All analyses were carried out with a GraphPad Prism 6.0 statistical program (GraphPad software, San Diego, CA, USA). A value p < 0.05 was considered statistically significant.

### Study Approval

Research and data collection protocols were approved by the Institutional Review Boards (Comitato Etico di Area 1) (prot. 20100005334) and by IRCCS Policlinico San Matteo Foundation Hospital (prot.20200046007). Written informed consent was obtained by all conscious patients in accordance with the Declaration of Helsinki. For unconscious patients next of kin were informed about all procedures and the inform consent was obtained from patients after recovery; for non-survivors was waived in accordance with the Italian law (Decreto legislativo 211/2003 art 5) (13).

## Results

### BAL of Severe COVID-19 Patients Are Enriched in NETs and Lung Biopsies in COVID-19 Deceased Patients Show EMT Pattern

**Figure 1:**
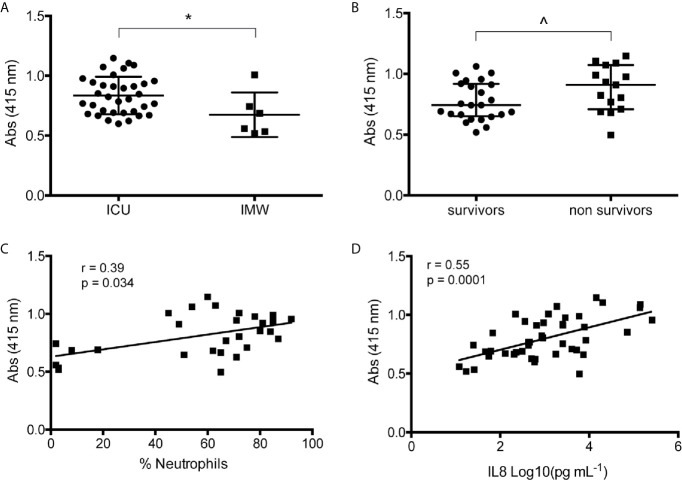
Analysis of BAL COVID-19 patients. **(A)** Quantification of NETs in BAL of mild (IMW) and severe (ICU) patients. NETs were measured by free-DNA quantification reading absorbance at 415 nm. **(B)** Quantified NETs were compared dividing samples in survivors and non-survivors. **(C)** Correlation analysis between NETs and percentage of neutrophils counted in the respective BAL sample. **(D)** Correlation analysis between NETs and IL8 quantified in the respective BAL sample. Data are represented as median (IQR). *p < 0.05; ^p = 0.05. r = Spearman coefficient.

In agreement with recent papers (8, 14–16), measuring NETs in BAL of mild (IMW) and severe (ICU) patients, we found that ICU have a significant higher amount of NETs compared to IMW patients (**Figure 1A**). Interestingly, when patients were divided in survivors and non-survivors, non-survivors group presents more NETs compared to patients who survived (**Figure 1B**). Finally, we assessed a significant direct correlation between NETs and neutrophil counts (**Figure 1C**), as well as the levels of IL8 (**Figure 1D**), most known cytokine related to neutrophil recruitment. **Figure S1** are representative images of immunohistochemical analysis of BAL of severe COVID-19 patients showing that the major cellular components are neutrophils, macrophages, epithelial cells and cellular/nuclear debris.

**Figure 2:**
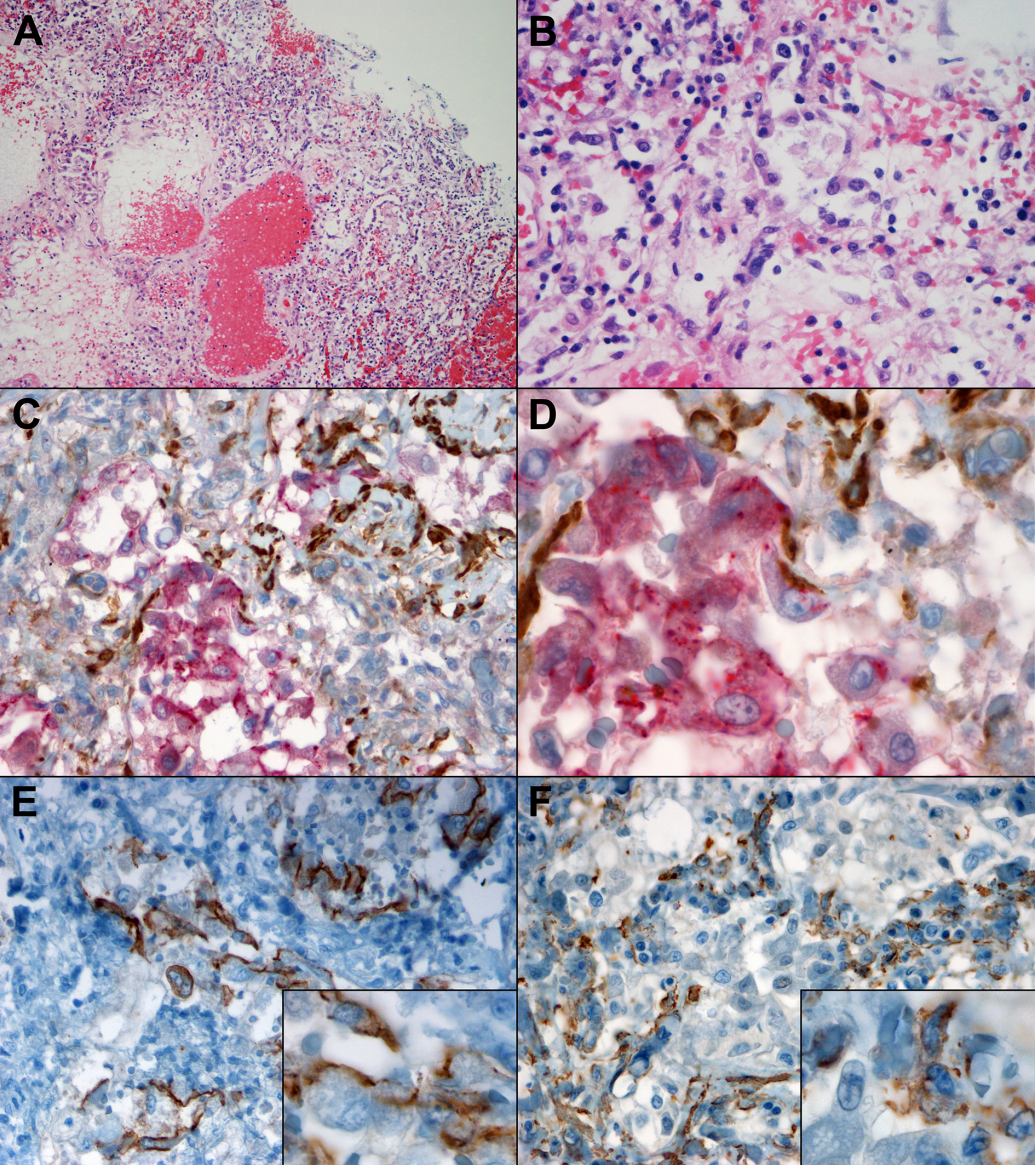
Morphological and immunohistochemical clues of EMT in lung tissue of patients which died of COVID-19, with DAD in proliferative phase. **(A)** At low power, oedema, vascular congestion, hemorrhage, and inflammatory infiltrates are visible (H&E, 10x). **(B)** At higher magnification, hyperplastic pneumocytes, a few granulocytes and fibrin deposition are evident (H&E, 40x). **(C)** Double IHC for an epithelial marker, CK7 (red) and a mesenchymal marker, α-SMA (brown) demonstrated co-expression in a subset of alveolar reactive pneumocytes (40x). **(D)** At higher magnification, a gradient of CK7 loss can be observed within the epithelial cells, and a subset co-expresses CK7 (red) as well as α-SMA (brown) (100x). **(E)** IHC for E-cadherin highlights partial loss in epithelial cells, some of which demonstrate a spindle morphology (inset) (40x, inset 100x). **(F)** IHC for α-SMA demonstrated the presence within epithelioid cells (40x, inset 100x).

The histopathological examination of lung tissues obtained from two patients who died for COVID-19 pneumonia demonstrated exudative and proliferative features of diffuse alveolar damage, corresponding to an early/intermediate phase of the disease (17). Moreover, a subset of pneumocytes is double positive for epithelial and mesenchymal markers, CK7 and α-SMA, respectively (**Figure 2**), suggesting establishment of lung fibrosis through EMT mechanism.

### NETosis Induces the EMT in an Alveolar Epithelial Cell Line

**Figure 3:**
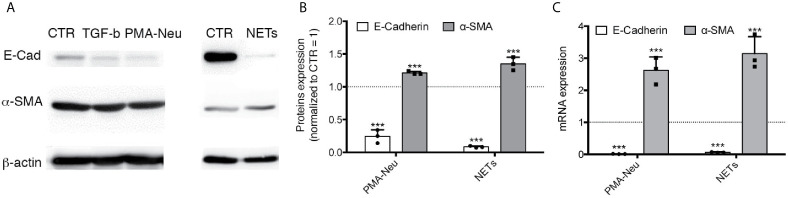
**(A)** Representative immunoblots of A549 treated with different conditions for 48 h. Membrane was immunodecorated with antibodies specific for E-Cadherin, α-SMA and β -actin. **(B)** Quantification of immunoblots of A549 using anti-E-cadherin or anti-α-SMA incubated with 2.5 x 10^6^ PMA-Neu or NETs isolated from 2.5 x 10^6^ PMA-Neu after 24 h. **(C)** RT-PCR analysis of RNA extracted by A549 treated with PMA-Neu or NETs for 24 h. Data are represented as mean ± SD of three independent replicates. ***p < 0.001 *vs*. CTR.

To demonstrate a direct relation between NETs induced by SARS-CoV2 infection and EMT mechanism, we firstly assess if NETs are able to induce EMT in a monoculture of A549, an alveolar epithelial cell line. To induce NETosis we used (2, 5) (9)PMA (**Figure S2**) and, as expected, NETs are visible as web-like structures and they are positive for the histone H3 (**Figure S2K**), HNE (**Figure S2N**) and MPO (**Figure S2R**), confirming literature data (18). Moreover, DAPI labeling further confirms that these proteins are associated to DNA (**Figures S2L, O, S**). Then, we treated A549 with 2.5 x 10^6^ neutrophils activated with PMA or with pure NETs isolated from the same amount of PMA-activated neutrophils. We evaluated the expression of two major proteins modified in EMT process: α-SMA (a mesenchymal marker) and E-cadherin (an epithelial marker). Western blot and RT-PCR analysis showed that α-SMA expression was significant up-regulated by PMA-Neu and NETs after 24 h compared to control cells (**Figure 3**), as well as TGF-β treatment (**Figure S3B**). Regarding E-Cadherin, PMA-Neu and NETs significantly downregulated protein and mRNA expression after 24 h of treatment (**Figure 3**), an effect that was similar to those obtained by TGF-β treatment (**Figure S3B**). Optical images confirmed that the modulation of proteins expression leads to a change in cell morphology (**Figure S3A**).

Knowing that NETosis can induce also cytotoxicity we evaluated A549 cell death after 24 h of treatment with PMA-Neu and NETs. The amount of cell death was assessed by PI staining acquired by flow cytometer. Analysis showed that PMA-Neu induced 33.70 ± 4.37% of cell death compared to control cells, in contrast to NETs (**Figure S3C**). To exclude that cytotoxicity observed after PMA-Neu treatment was due to the presence of PMA, we evaluated also PMA effect on A549 viability demonstrated that PMA induce only 8.25 ± 0.97% of cell death (**Figure S3C**).

### Airway *In Vitro* Model

Assessed that NETs are present at high level in BAL of severe COVID-19 patients and that they are able to induce EMT, we next set-up an airway *in vitro* model to connect SARS-CoV2 infection, production of NETs and EMT trigger. To make airway *in vitro* model we cultivated A549 cells at ALI, AM were seeded at the apical side of A549 cells, while neutrophils were added to the basolateral chamber of the transwell to mimic alveolar microenvironment (19, 20). Our *in vitro* model was then inoculated with SARS-CoV2 (Neu+AM+SARS-CoV2). As a control, A549 cells were cultured alone, or with SARS-CoV2, or with Neu+SARS-CoV2, or with AM+SARS-CoV2.

**Figure 4:**
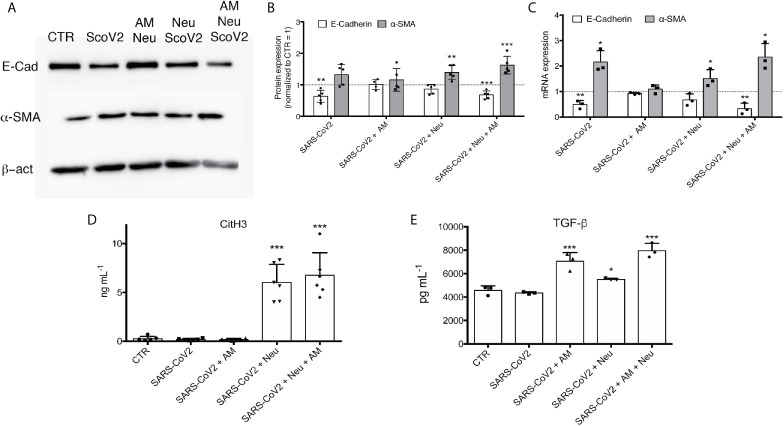
**(A)** Representative immunoblots of A549 treated with different conditions for 48 h. Membrane was immunodecorated with antibodies specific for E-Cadherin, α-SMA and β -actin. **(B)** Semiquantitative analysis of immunoblots and **(C)** RT-PCR analysis of airway *in vitro* model cultured with SARS-CoV2, AM+SARS-CoV2, Neu+SARS-CoV2 or Neu+AM+SARS-CoV2 after 48 h of treatment. **(D)** NETs quantification by CitH3 ELISA. **(E)** TGF-β quantification by ELISA assay. Data are represented as mean ± SD. ***p < 0.001 *vs*. CTR; *p < 0.05 *vs*. CTR; **p < 0.01 *vs*. CTR.

After 48 h of incubation, protein (**Figures 4A, B**) and mRNA expression analysis (**Figure 4C**) showed that the only condition that showed a complete expression pattern of EMT was Neu+AM+SARS-CoV2. In fact, even if α-SMA (mRNA and protein level) is significant up-regulated also in Neu+SARS-CoV2 condition, we did not see a down-regulation of E-cadherin. For SARS-CoV2 condition, even if at mRNA we observed a significant modulation of mRNA of α-SMA and E-Cadherin (**Figure 4C**), at protein level we did not see the same significance (**Figure 4B**).

To clarify if NETs are produced by neutrophils and if they are possibly involved in triggering EMT, we quantified NETs showing that there is high concentration of NETs in Neu+SARS-CoV2 and Neu+AM+SARS-CoV2 compared to control cells (**Figures 4D**, **S4C**). As expected, in SARS-CoV2 and AM+SARS-CoV2 we did not find any sign of NETs given the absence of neutrophils (**Figures 4D**, **S4C**).

Quantifying TGFβ (**Figure 4E**), most known pro-fibrotic factor, IL8 (**Figure S4A**) and IL1β (**Figure S4B**), the two major cytokines involved in inducing NETosis (21), we observed that they are present only in the treatment condition where are present AM (Neu+AM+SARS-CoV2 and AM+SARS-CoV2). However, assessed that in AM+SARS-CoV2 there is no EMT induction (**Figures 4A–C**), we can suggested that in this *in vitro* model are necessary the presence of all factors mentioned to have a complete EMT trigger.

All the results obtained with A549 cell line, was confirmed by using another cell type to perform *in vitro* model, 16HBE, human bronchial epithelial cell line that express ACE2 (22), in contrast to A549 that did not express the specific receptor for SARS-CoV2 (**Figure S5**).

## Discussion

This study allows us to understand that neutrophils can play a pivotal role in inducing efficiently EMT through NETosis, an observation that, at the best of our knowledge, has never been demonstrated before in the lung context and, more importantly, in severe COVID-19. NET-related injuries are usually studied in the context of the endothelium (21, 23). In particular, the prothrombotic role (24) of NETs and their ability to drive endothelial-mesenchymal transition (25) has been thoroughly analyzed. However, recent reports suggested that the infiltration of neutrophils in the alveolar space interferes with cell-cell adhesion of lung epithelial cells through HNE (26). Moreover, neutrophils are able to promote EMT by releasing TGF-β, neutrophil gelatinase-associated lipocalin (NGAL) (27) or Proteinase-activated receptor 4 (PAR4) (28). Herein, we demonstrate that NETs can also directly activate the EMT program in A549 cells, a commonly used model of type II pneumocytes. We showed that the addition of purified NETs to A549 cells induced a significant overexpression of the mesenchymal marker α-SMA after 24 h of treatment, together with a decrease of E-cadherin expression (**Figure 3**). Comparing the effect exerted by neutrophils activated with PMA (PMA-Neu) and pure NETs on A549, we found that although both treatments are able to trigger the EMT (**Figure 3**), PMA-activated neutrophils are able to induce also death of A549 cells (**Figure S3C**). This cytotoxicity could be due to the presence of other factors secreted by neutrophils induced by PMA, that are absent in purified NETs preparation. In any case, these results shed new light on the mechanism that leads to the EMT in the lung: exaggerated NETosis not only induces tissue damage (2, 5, 6) but it can also play a crucial role in triggering EMT of pneumocytes.

High concentration of NETs was detected in the peripheral blood and tracheal aspirates of SARS-CoV2-infected patients correlating significantly with disease severity (7, 8, 14), together with an exaggerated infiltration of neutrophils in alveolar spaces, highlighting the crucial role of these phagocytes in the pathogenesis of COVID-19 (29, 30). Here, we confirmed and expanded previous observations by quantifying NETs in the alveolar micro-environment. We took into consideration two groups of patients, those with mild disease (patients that were admitted to the hospital with signs and symptoms of bilateral interstitial pneumonia but did not required intubation) and those with severe disease (who needed ICU support). Our data demonstrated higher levels of NETs in severe patients compared to IMW (**Figure 1A**); moreover, NETs correlated with the percentage of neutrophils in BAL (**Figure 1C**), as well as with the levels of IL8 (**Figure 1D**), the most common chemoattractant of neutrophils and inducer of NETosis.

Observing this direct relation between NETs and COVID-19 severity (**Figure 1B**) and their potential role in inducing EMT (**Figure 3**), we attempted to recreate the alveolar space by an *in vitro* model to further study what happen when SARS-CoV2 is added. Interestingly, we assessed that only the Neu+AM+SARS-CoV2 culture condition showed a complete mRNA and protein expression pattern related to EMT; in fact, α-SMA was significantly up-regulated, in contrast to E-cadherin that was decreased by the treatment compared to control cells (**Figures 4A, B**). In line with Stewart et al., even if we observed that only SARS-CoV2 can modulate both α-SMA and E-Cadherin mRNA expression, this effect was not visible at protein level. Also Stewart et al. demonstrated that treating A549 with SARS-CoV2 there was only an upregulation of the *ZEB1* gene together with the downregulation of *EPCAM* without observing a protein expression alteration (31). Interesting are the results obtained in Neu+SARS-CoV2 and AM+SARS-CoV2. In fact, even if we observed the same production of NETs in Neu+SARS-CoV2 and Neu+AM+SARS-CoV2 (**Figure 4D**), and the same release of IL8, IL1β and TGF-β in AM+SARS-CoV2 and Neu+AM+SARS-CoV2 (**Figures S4A, B**, **4E**), we did not see a protein and mRNA expression modulation towards EMT, neither in Neu+SARS-CoV2 nor in AM+SARS-CoV2 (**Figures 4A–C**). These results suggest that in this experimental condition, that mimic the alveolar environment, the presence of all factors mentioned above are necessary to have a complete induction of EMT in alveolar epithelial cells.

We are aware about the low endogenous expression by A549 of ACE2, the specific receptor for SARS-CoV2 cell entry (32, 33), reducing the potential effect of SARS-CoV2 on our experimental setting. However, we made the same *in vitro* model using 16HBE (**Figure S5**), a cell line expressing ACE2, confirming the results obtained from A549.

What we particularly propose thanks to our model is that macrophages, by releasing IL8 and IL1β, two cytokines significantly increased in the plasma (34, 35) and BAL-fluid of severe COVID-19 patients (10), potentiate the capacity of neutrophils to release NETs amplifying the noxious activity on pneumocytes, favoring the EMT process. Our data are sustained by previous studies that showed the existence of a feedback loop between neutrophils/NETs and release of IL8 (36) and IL1β (6).

Last but not least, thanks to immunohistochemistry analysis we observed that a subset of pneumocytes present both epithelial marker (CK7) and mesenchymal marker (α-SMA) (**Figure 2**), thus confirming that epithelial cells acquired a mesenchymal phenotype, supporting our *in vitro* findings.

In conclusion, this study highlights the contribution of neutrophil activation towards NETosis in the induction of EMT. This knowledge is very important, not only for COVID-19, but also for other lung diseases, such as autoimmune diseases and chronic lung graft rejection that have been associated at various degrees with alveolar and small airway influx of neutrophils and their activation (37, 38); so, our results could be useful in those clinical areas that involved neutrophils activation towards NETosis. Furthermore, we are the first demonstrating with an alveolar *in vitro* model that NETosis-induced EMT should be considered as an important pathogenic step of lung fibrosis consequent to neutrophilic inflammation after SARS-CoV2 infection, confirmed by lung biopsies of COVID-19 deceased patients. Our findings also support literature suggesting as future therapeutic interventions the modulation of neutrophils activation.

## Data Availability Statement

The original contributions presented in the study are included in the article/**Supplementary Material**. Further inquiries can be directed to the corresponding author.

## Ethics Statement

The studies involving human participants were reviewed and approved by Institutional Review Boards (Comitato Etico di Area 1) (prot. 20100005334) and by IRCCS Policlinico San Matteo Foundation Hospital (prot.20200046007). The patients/participants provided their written informed consent to participate in this study.

## Author Contributions

LP, FM, and DL designed the research. LP, VF, SB, EP, and EG performed the *in vitro* experiments. MD’A, MM, SB, FV, and LP handled BAL samples. TF, RC, and LS collected BAL from COVID-19 patients. MV and AL performed western blot analysis. MA quantified IL1beta. GL, MN, and LC performed immunohistochemistry analyses on COVID-19 biopsies. LP and VF analyzed data. LP, SB, VF, and FM wrote the main text. MG, VC, PB, FB, and DL revised the manuscript. All authors contributed to the article and approved the submitted version.

## Funding

Fondazione Cariplo (COVIM project); Ministry of Health funds to IRCCS Foundation Policlinico San Matteo Grant (RC) and Ministry of Health funds COVID-2020-12371760​. The funders had no role in study design, data collection and analysis, or preparation of the manuscript.

## Conflict of Interest

The authors declare that the research was conducted in the absence of any commercial or financial relationships that could be construed as a potential conflict of interest.
