# Supplementary material for: Neutrophil Extracellular Traps Induce the Epithelial-Mesenchymal Transition: Implications in Post-COVID-19 Fibrosis
Source: Front Immunol. 2021 Jun 14;12:663303. doi: 10.3389/fimmu.2021.663303 (PMC8236949; doi:10.3389/fimmu.2021.663303)
Supplement: Supplementary file 1 [file DataSheet_1.docx]

Supplementary Material


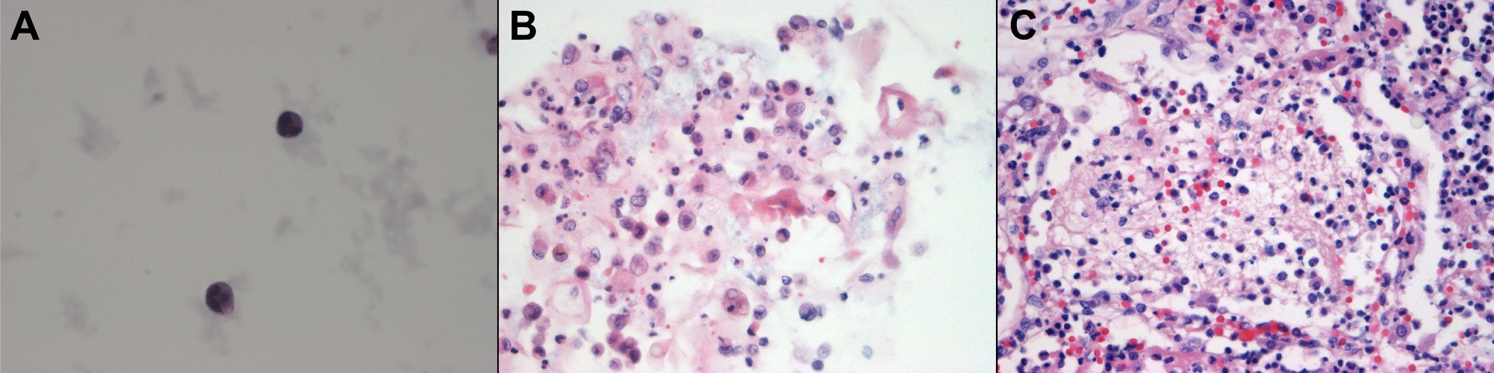


**Supplementary Figure 1.** Morphological evaluation of BAL samples. (A) Two neutrophils in the cytological smear; OM 100x. (B) Cell block characterized by macrophages, epithelial cells, neutrophils and cellular/nuclear debris; H&E OM 40x. (C) Histological evaluation of lung tissue with macrophages, epithelial cells, neutrophils and cellular/nuclear debris in alveolar spaces; H&E OM 40x.


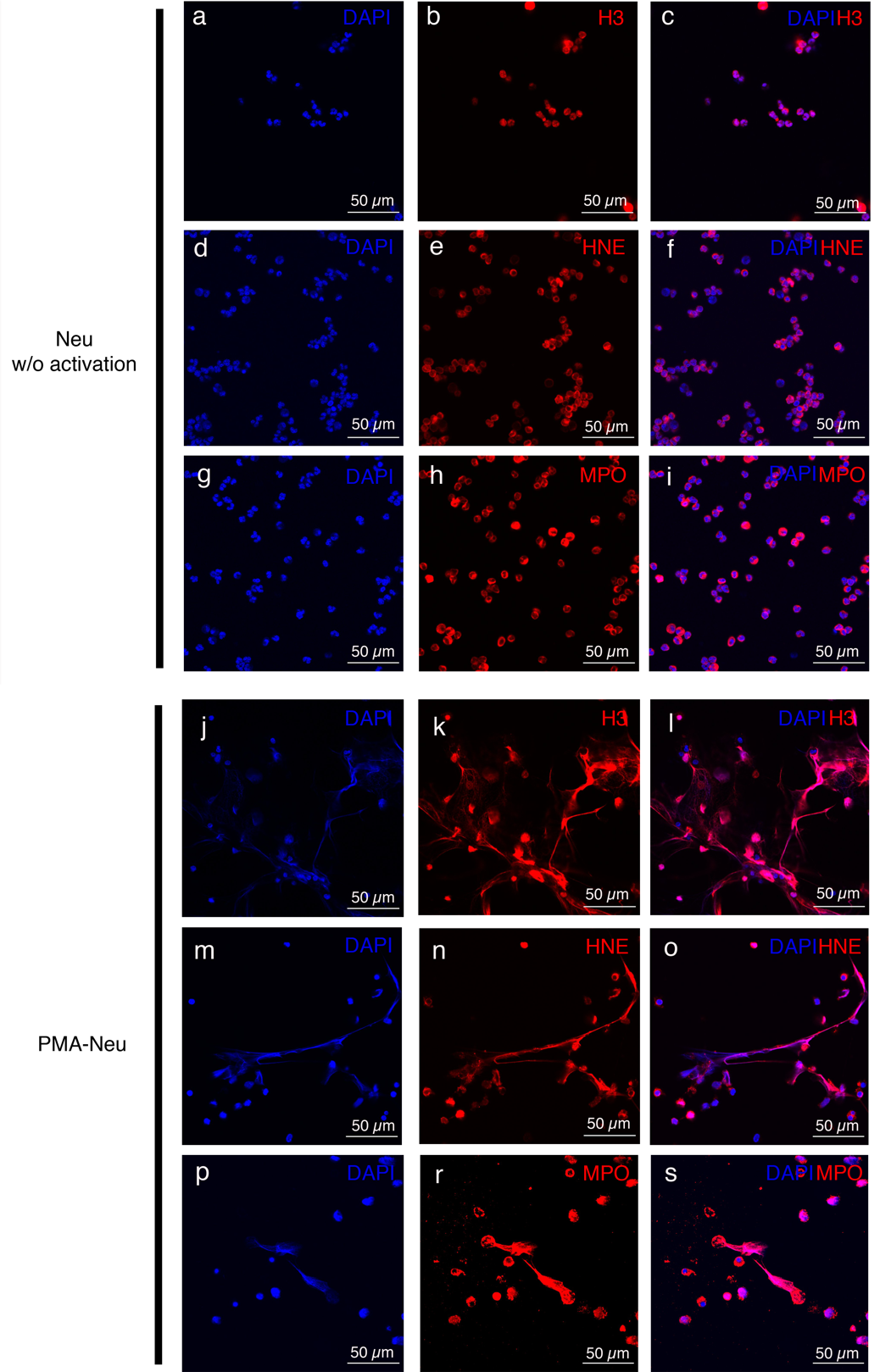


**Supplementary Figure 2.** Confocal images of neutrophils activated or not towards NETosis. (A-I) Neutrophils isolated from peripheral blood of healthy donors without any treatment or (J-S) treated with 100 nM of PMA for 4 h. All samples were then labeled with (B,K) anti-H3 or (E,N) anti-HNE or (H,R) anti-MPO Ab (red signal). (A,D,G,J,M,P) DAPI (blue signal) was used to label DNA. (C,F,I,L,O,S). Images of merged channels. Scale bar = 50 µm.

**
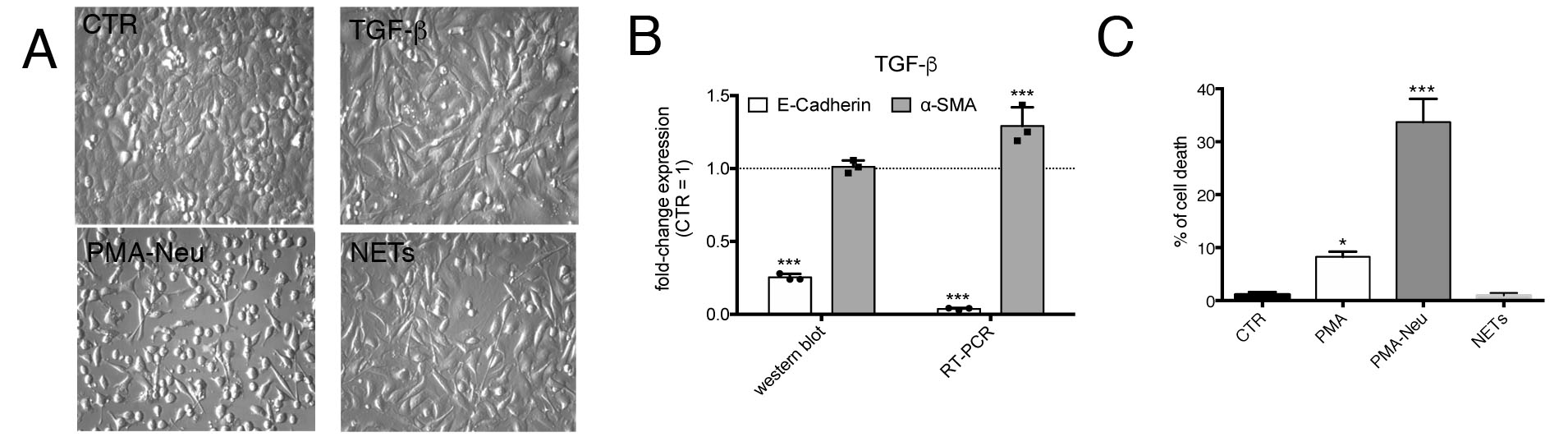
**

**Supplementary Figure 3.** (A) Representative optical images of A549 incubated with TGF-β, 2.5 x 10^6^ PMA-Neu and pure NETs isolated from 2.5 x 10^6^ PMA-Neu. Images were acquired with a 40X magnification. (B) Quantification of immunoblots and RT-PCR analysis of A549 treated with TGF-β for 24 h. (C) Cell death analysis of A549 incubated with 100 nM PMA, 2.5 x 10^6^ PMA-Neu or NETs (24 h) was evaluated by flow cytometry labeling A549 with PI before the acquisition. Data are represented as mean ± SD of three independent replicates. ***, p<0.001 vs. CTR; *, p<0.05 vs. CTR.


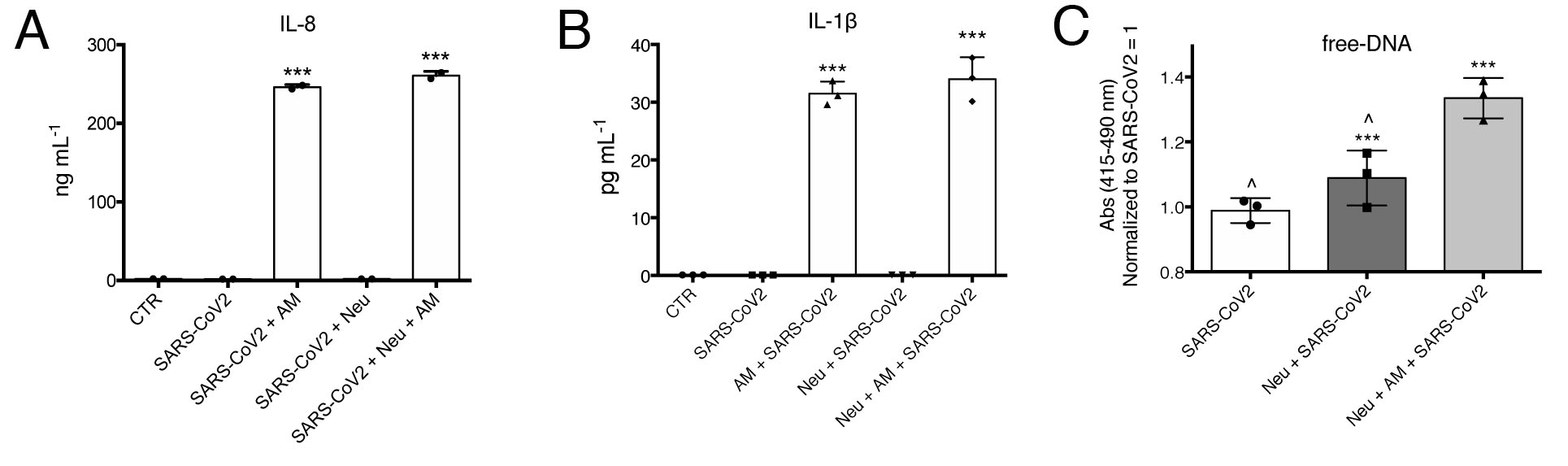


**Supplementary Figure 4.** (A) IL8 and (B) IL1β quantification in culture media of A549 *in vitro* airway model by ELISA assay. (C) free-DNA quantified in culture media of A549 *in vitro* airway model. Data are represented as mean ± SD. ***, p<0.001 vs. CTR; ^, p<0.001 vs. SARS-CoV2.

**
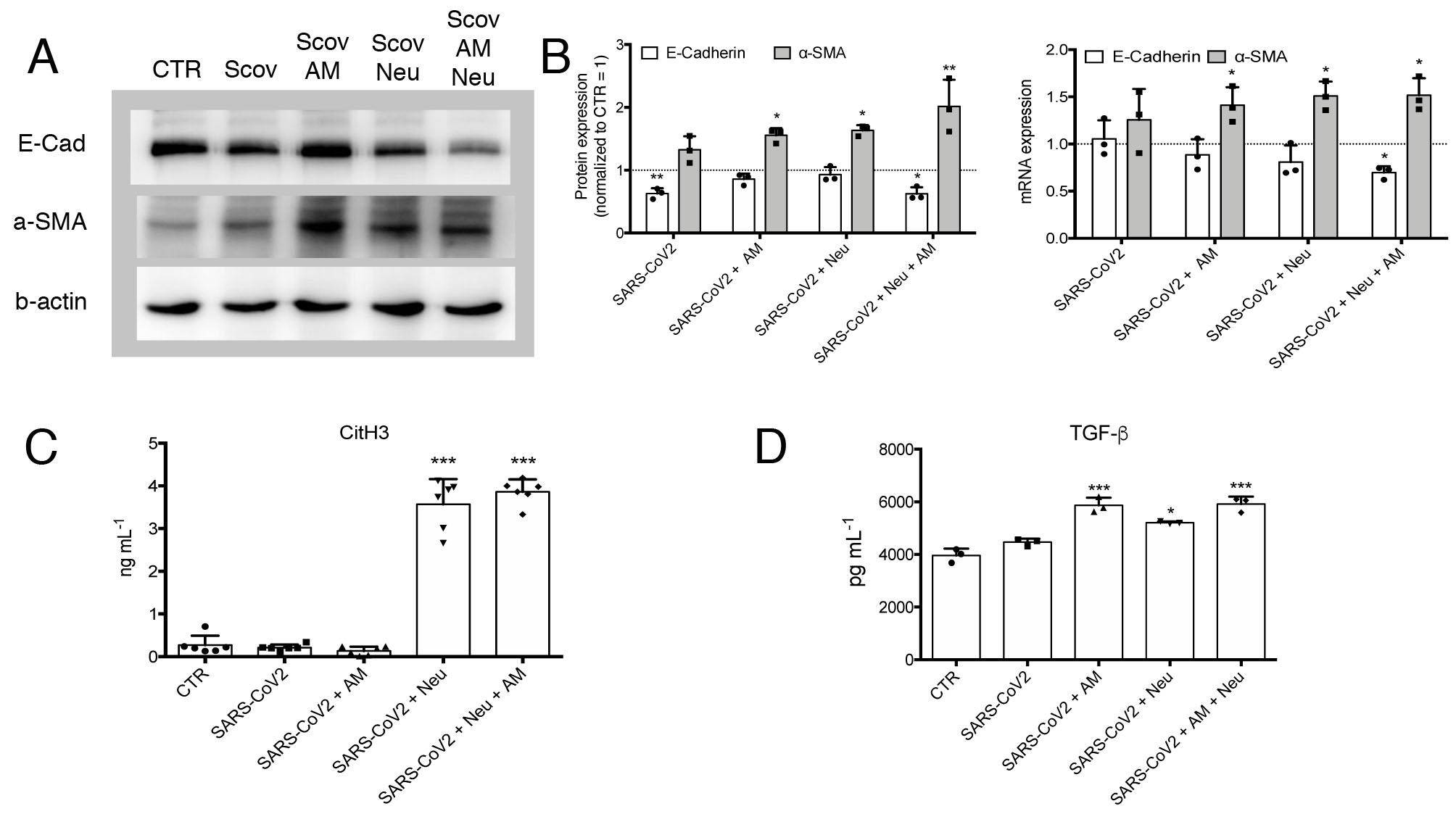
**

**Supplementary Figure 5.** (A) Representative immunoblots of 16HBE incubated with SARS-CoV2, AM+SARS-CoV2, Neu+SARS-CoV2, or Neu+AM+SARS-CoV2 for 48 h. (B) Quantification of immunoblots and RT-PCR analysis after 48 h of treatment. (C) CitH3 quantification in cell culture medium of *in vitro* model. (D) TGF-β quantified in culture media of 16HBE *in vitro* airway model. Data are represented as mean ± SD. ***, p<0.001 vs. CTR; *, p<0.05 vs. CTR.
